# Thioredoxin-Related Transmembrane Proteins: TMX1 and Little Brothers TMX2, TMX3, TMX4 and TMX5

**DOI:** 10.3390/cells9092000

**Published:** 2020-08-31

**Authors:** Concetta Guerra, Maurizio Molinari

**Affiliations:** 1 Institute for Research in Biomedicine, Faculty of Biomedical Sciences, Università della Svizzera italiana (USI), 6500 Bellinzona, Switzerland; 2 School of Life Sciences, École Polytechnique Fédérale de Lausanne, 1015 Lausanne, Switzerland

**Keywords:** endoplasmic reticulum, ERAD, folding, PDI, TMX

## Abstract

The endoplasmic reticulum (ER) is site of synthesis and maturation of membrane and secretory proteins in eukaryotic cells. The ER contains more than 20 members of the Protein Disulfide Isomerase (PDI) family. These enzymes regulate formation, isomerization and disassembly of covalent bonds between cysteine residues. As such, PDIs ensure protein folding, which is required to attain functional and transport-competent structure, and protein unfolding, which facilitates dislocation of defective gene products across the ER membrane for ER-associated degradation (ERAD). The PDI family includes over a dozen of soluble members and few membrane-bound ones. Among these latter, there are five PDIs grouped in the thioredoxin-related transmembrane (TMX) protein family. In this review, we summarize the current knowledge on TMX1, TMX2, TMX3, TMX4 and TMX5, their structural features, regulation and roles in biogenesis and control of the mammalian cell’s proteome.

## 1. Introduction

About one third of the proteome in eukaryotic cells is made of membrane and secretory proteins [1]. Their production and maturation occurs within the ER with help and under surveillance of resident chaperones and folding enzymes, such as the members of the PDI family [2]. PDIs assist protein folding by catalyzing the formation of the native set of intra- and inter-molecular disulfide bonds (oxidation); they can also correct structural errors by disassembling non-native disulfides to promote their conversion into the native set (isomerization) [3,4]; they can facilitate the translocation across the ER membrane of terminally misfolded polypeptides by dissolving intra- and inter-molecular disulfide bonds (reduction), in a step that precedes their degradation by cytosolic 26S-proteasomes [5,6]. In addition to these activities, PDIs can also act as regulators of the luminal calcium homeostasis [7] and participate to multimeric structures such as the prolyl 4-hydroxylase [8] or the oligosaccharyltransferase complexes [9].

**Figure 1:**
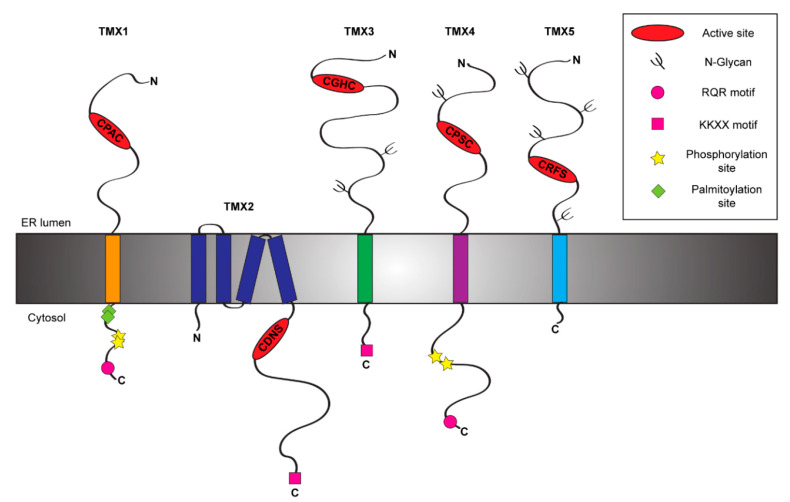
Schematic representation of the TMX protein family members. The figure shows the topology and the main structural and functional features of the five members of the TMX family [12,13,14,15,16].

More than 20 PDI family members have been identified, so far [10]. The reasons for such a high number is not fully understood. However, their tissue distribution, membrane topology, and organization of the active site hint at client-specificity and high functional versatility [11]. Most PDI family members are soluble in the ER lumen, with few membrane-anchored exceptions [4]. The TMX protein family comprises five membrane-tethered PDIs (TMX1, TMX2, TMX3, TMX4 and TMX5) [12,13,14,15,16] (Figure 1 and Table 1). These proteins are all characterized by an N-terminal signal sequence for ER targeting and one catalytically active thioredoxin (TRX)-like domain (known as type-a TRX-like domain), containing the active site. TMX1, the best characterized member of the TMX family, preferentially interacts with membrane-bound folding-competent and folding-defective clients [17,18]. In contrast, the other members of the family have been poorly studied, if at all. 

## 2. TMX1: A Topology-Specific ER-Resident Reductase

TMX1 (other name TXNDC1) is the best-known member of the TMX family. It has been identified in 2001 by Matsuo and colleagues [16] among the genes up-regulated by TGF-β [19]. TMX1 is a single-pass type I protein of 280 residues with a large luminal N-terminal region harboring a TRX-like domain and a short cytosolic tail [16] (Figure 1). TMX1 displays a di-arginine motif that ensures its retention within the ER [16,20,21] (Figure 1). The cytosolic tail of TMX1 also contains both palmitoylation [22] and phosphorylation sites [23] (Figure 1). These modifications affect the sub-ER localization of TMX1 and may determine the spectrum of clients [21]. TMX1 is ubiquitously expressed in human tissues, with the highest levels in kidney, lungs, placenta and liver [16]. Unlike other members of the PDI family, *TMX1* does not contain an ER stress responsive element (ERSE) within its promoter region [24] and indeed it is not up-regulated upon ER stress [25]. Deletion of the *TMX1* gene is innocuous at the cellular level. This suggests the activation of compensatory mechanisms in cultured cells, where other members of the PDI family may play a role [26]. At the organism level, however, the absence of TMX1 has consequences as it increases susceptibility to liver damage in mice challenged with lipopolysaccharides [26]. From a functional point of view, TMX1 displays a non-canonical CPAC active site in its type-a TRX-like domain [16] (Figure 1 and Table 1). The proline in position 2 suggests a role as reductase [27], since it destabilizes the disulfide state and favors the di-thiol reduced form of the active site [4,28]. Consistently, TMX1 is predominantly reduced in vivo [25], and in vitro it reduces insulin disulfides [16]. Of additional support to the putative function of TMX1 as an ER reductase, it has been demonstrated that TMX1 overexpression enhances the cytotoxicity of the toxins ricin and abrin, two type 2 ribosome-inactivating proteins requiring a step of reduction in the ER before the dislocation of the catalytic subunits to the cytosol [29]. TMX1 has been also found to interact with vitamin K epoxide reductase (VKOR), an enzyme involved in the process of blood coagulation working with membrane-tethered TRX-like proteins, which serve as redox partners [30].

### 2.1. TMX1 Assists Folding of Membrane-Tethered Polypeptides

The formation of the correct pattern of disulfide bonds represents a rate-limiting step during protein folding and the assistance of PDIs for their formation is of crucial importance [4]. The identification of PDIs clients has taken advantage from the exploitation of trapping mutant variants of the individual PDIs [31]. Here, the mutation of the C-terminal cysteine within the PDI’s active site stabilizes the mixed disulfide reaction-intermediate thereby “trapping” client proteins in a covalent complex. This approach has been instrumental to identify TMX1 clients [17]. Interactomic analysis of the TMX1 trapping mutant obtained by mutation of the CPA**C** active site to its CPA**A** trapping version, revealed the preference towards endogenous membrane-tethered cysteine-containing proteins [17]. The preference of TMX1 for membrane-bound clients and its competence in facilitating their conformational maturation have been confirmed using ectopically expressed model client proteins. The role of TMX1 in biogenesis of membrane-tethered polypeptides is supported by formation of a functional complex with the ER lectin Calnexin (CNX) [17,25]. CNX is a single-pass type I molecular chaperone that associates with TMX1 via transmembrane anchors and engages newly synthesized polypeptides upon association with their mono-glucosylated N-linked glycans [32]. Interestingly, the engagement of client substrates stabilizes the formation of the complex between TMX1 and CNX [17]. As such, TMX1 recruits membrane-bound N-glycosylated clients through a cooperative interaction with the ER lectin CNX intervening during their folding.

### 2.2. TMX1 Selectively Intervenes in ERAD of Membrane-Tethered Folding-Defective Polypeptides

The reduction of disulfide bonds in terminally misfolded polypeptides is a crucial preparative step preceding their dislocation across the ER membrane for degradation by cytosolic 26S proteasomes [5,6,33]. TMX1 has recently been added to the list of the PDIs contributing to ERAD of misfolded proteins [18]. Its reported role in the retro-translocation of catalytic toxin subunits across ER membrane [29] as well as its reductive potential [20,25] firstly pointed out a possible implication of TMX1 in ERAD. Consistently, the expression of the trapping mutant version of TMX1 delayed clearance of faulty, membrane-bound gene products from the ER [18]. Disposal of the same misfolded ectodomains detached from the membrane remained unaffected upon TMX1 inactivation [18] revealing TMX1 as a reductase involved in protein folding [17] and degradation [18] with strong preference for membrane-anchored polypeptides. Of note, TMX1 preferential association with membrane substrates displaying different transmembrane regions suggests that the characteristics of the transmembrane anchors do not represent a discriminant factor for substrate selection in both folding and ERAD pathways. These characteristics qualify TMX1 as the first example of redox-catalyst within the PDI family that selects ERAD clients based on their topology [17,18].

### 2.3. MAM-Localized TMX1 Acts as a Regulator of Ca^2+^ Flux

Research from the past decade demonstrated that many ER chaperones and folding enzymes are multifunctional proteins, whose activities go beyond their roles in protein folding and quality control [34]. Indeed, in addition to its role as topology-selective reductase in the folding and degradation pathways [17,18], TMX1 also regulates Ca^2+^ flux between ER and mitochondria [35]. This function relies on the localization of TMX1 at ER-mitochondria contact sites, aka MAM (mitochondria-associated membranes), which is regulated by palmitoylation of two cytosolic membrane proximal cysteines [22] (Figure 1). At MAM, TMX1 interacts with SERCA2b, a Ca^2+^-ATPase responsible for the calcium flux from the cytosol to the ER lumen [36]. This interaction inhibits the ion transport function of SERCA2b, determining an increase in cytoplasmic Ca^2+^, which activates Ca^2+^ import in mitochondria to stimulate the oxidative phosphorylation [36]. The mechanism of TMX1 action in this pathway is poorly understood. However, the association of TMX1 with SERCA2b relies on TMX1’s enzymatic activity, since it is abolished by mutations within the TRX-like domain and under reducing conditions, whereas it is enhanced upon chemically induced hyperoxidation [36]. Possibly, TMX1 reduces a luminal disulfide bond within the SERCA2b structure, thus regulating the function of the pump [36,37]. Consistent with its role as calcium flux regulator, *TMX1* silencing hampers formation of ER-mitochondria contact sites, a prerequisite for ER to mitochondria calcium transfer [35,36].

From a physiological point of view, TMX1 levels and TMX1-regulated calcium homeostasis impact on tumor growth and in vivo studies reveal a tumor-protective role for TMX1 [36]. Indeed, reduced levels of TMX1 have been found upon breast cancer progression. In this context, cells expressing low levels of TMX1 display a reduced mitochondrial metabolism, which confers resistance to the effect of mitochondrial poisons [36] that are currently used as treatment for many cancers [38]. Additionally, TMX1 expression has been also shown to be protective against oxidative stress in Schwann cells, and it is enhanced upon cells treatment with Vitamin C [39].

## 3. TMX2 and its Cytosolic Active Site

Among TMX family members, TMX2 (alternative name TXNDC14) undoubtedly is the most mysterious. It is a non-glycosylated protein of 296 amino acids, which has been identified in 2003 upon cloning from a fetal cDNA library [40]. The topology of TMX2 has been only recently characterized. Initially, it has been described as a type I membrane protein [40]. A recent study clarified its topology showing that TMX2 is a multi-spanning protein displaying both the N- and the C-terminal regions within the cytosolic side [12] (Figure 1). As such, the peculiar SNDC catalytic site of TMX2 is oriented towards the cytosol [12], in contrast with the other members of the TMX family (Figure 1). The long C-terminal tail of TMX2 contains a canonical ER retention signal (-KKDK) [40] (Figure 1). TMX2 is localized in different ER sub-compartments, such as in the nuclear outer membrane [12], or at the MAM [22]. TMX2 expression is ubiquitous with the highest levels in brain, heart, liver, kidney and pancreas [40]. Moreover, TMX2 is up-regulated upon oxidative stress, but not upon hypoxia, heat shock, or ER stress, consistently with the lack of an ERSE motif within its promoter region [12]. *TMX2* gene deletion in mice is embryonic lethal, implying a crucial role of this protein at early stages of development [41].

So far, no information is available on the physiologic function of TMX2 (Table 1) with the exception of a possible participation of TMX2 in the importin-β:Ran complex that controls nuclear targeting of select cargo proteins [12,42]. The localization of TMX2 in the outer nuclear membrane, which is contiguous to the ER membrane, is crucial for the binding of the importin-β:Ran complex and the maintenance of the nucleocytoplasmic Ran protein gradient, whereas mutations in the TMX2 active site only partially impair them [12].

TMX2 can also localize at the MAM [22], where it forms a functional complex with CNX and the Ca^2+^-pump SERCA2 to modulate the calcium flux [41] in a function that mimics the one reported for TMX1 [36] and that could be exerted in tissue-specific manner.

Consistently with its high expression in brain tissue, missense mutations of *TMX2* have recently been associated with brain developmental abnormalities [41] and microlissencephaly [43], a rare congenital brain disorder [44]. This phenotype could result from a loss of the protective role of TMX2 from ER stresses [45], which represents an important concurrent cause of neuronal death [46].

## 4. TMX3, a Classic PDI

TMX3 is a single-pass type I glycoprotein of 454 amino acids (Figure 1), which has been identified in 2005 among the uncharacterized proteins containing the consensus sequence for a TRX-like domain [13]. This PDI family member, also known as TXNDC10, displays within its large N-terminal region two N-glycosylation sites (Figure 1) and three TRX-like domains, one catalytically active type-a domain followed by two inactive type-b (b and b′) domains [47]. Additionally, its C-terminal region contains a classical KKKD retention sequence [13] (Figure 1). TMX3 transcripts have been found in a great variety of tissues with the highest levels in heart and skeletal muscle [13]. Consistent with other TMX family members, *TMX3* does not contain an ERSE motif and it is not upregulated upon ER stress [13]. The type-a TRX-like domain of TMX3 is characterized by a canonical CGHC sequence [13] (Figure 1 and Table 1), which corresponds to the catalytic sequence of PDI [48]. TMX3 acts as an oxidase in vitro [13] and its b′ domain is possibly involved in substrate recruitment [47].

The precise role of TMX3 in cells has not been established, yet. Preliminary studies show a protective function of TMX3 against neuronal atrophy in mice models for Huntington’s disease [49], a progressive brain disorder caused by an inherited CAG trinucleotide repeat expansion in the *huntingtin* (*HTT*) gene [50]. The molecular basis of this protective effect is unclear, also because HTT is a cytosolic protein and a direct interaction with the functional portion of TMX3 can be ruled out. Since it has been shown that the expression of mutated HTT triggers ER stress, an hypothesis is that TMX3 protects cells against neuronal atrophy mitigating the stress situation [49].

Both deletion and missense mutations in *TMX3* gene have been linked to coronary artery diseases [51] and microphthalmia [52], a disease associated with retarded growth of the eye [53].

## 5. TMX4, the Paralogue of TMX1

TMX4 (alternative name TXNDC13) is a single-pass type I glycoprotein (Figure 1) of 349 amino acids that has been identified in 2010 during a database search for TRX-like domain containing proteins [14]. Phylogenetic analysis showed that TMX4 represents the paralogue of TMX1 [21], with whom it shares high similarity within the N-terminal luminal regions despite the presence of an N-glycosylation site [14,21] and a di-arginine RQR retention motif within the C-terminal domain [21]. Both proteins display two phosphorylation sites within the cytosolic domain [23] (Figure 1), which could modulate sub-ER localization upon recruitment of select binding partners [21]. TMX4 expression is ubiquitous with the highest levels in heart tissue [14]. Consistently with the lack of an ERSE motif within its promoter region, TMX4 is not up-regulated during ER stresses [14]. TMX4 has one luminal type-a TRX-like domain, which contains a non-canonical CPSC active site [14] (Figure 1 and Table 1). The proline in position 2 hints at a role as ER-reductase [27] and, indeed, TMX4 efficiently reduces insulin disulfides in vitro [14]. Additionally, also TMX4 works as VKOR redox partner, even if this interaction results weaker compared to the one established by VKOR and TMX1 [30].

From the functional point of view, no evidence has been reported on substrate preference and possible roles of TMX4 in cells, even though different hypothesis have been formulated. The structural similarities with TMX1 strongly hint at a role of TMX4 as ER reductase possibly acting in both protein folding and degradation pathways. Supporting an involvement in protein folding, TMX4 interacts with CNX and with ERp57 [14], a soluble member of the PDI protein family [54]. In this functional complex, TMX4 could enzymatically modify clients directly promoting their oxidative maturation, or it could indirectly contribute to protein folding by reducing the ERp57 catalytic site thus promoting its function as a glycoprotein-specific oxidase [14]. However, it should be considered that since TMX4 is an N-glycosylated protein, the interaction with CNX could be also due to the fact that TMX4 is itself a client of CNX during its folding. TMX4 does not interact with any of the known ERAD factors and its knockdown has no effect on the degradation rate of the α1-antitrypsin NHK variant [14], which is a well-characterized ERAD model substrate. Based on these evidences, a role of the reductase TMX4 in ERAD has been excluded [14]. Our opinion is that this conclusion should be re-examined by considering more recent findings revealing the topology-specific client selection of the TMX4 paralogue TMX1 [17,18] and that a possible role of TMX4 in protein quality control should be assessed by using membrane-tethered folding-competent and -incompetent model polypeptides. Finally, a recent study showed that TMX4 also distributes at the inner membrane of the nuclear envelope (NE) with the N-terminal portion facing the NE lumen [55]. Since many protein complexes resident at the NE (including the LINC:Torsin A complex) are modulated by redox cycles, a role for TMX4 in the regulation of the NE structure can be envisioned [55].

## 6. TMX5, a Natural Trapping Mutant Member of the TMX Family

TMX5 (TXNDC15) is another poorly characterized member of the TMX family. It is a predicted single-span type I protein of 360 amino acids (Figure 1). It has been identified in a large scale protein screening in 2003 [56]. Structurally, TMX5 has a large N-terminal luminal domain and a very short C-terminal cytoplasmic tail of 18 amino acids, which lacks canonical ER retention signals. This characteristic suggests that unlike other TMXs, TMX5 could potentially leave the ER and traffic through the secretory pathway. Unfortunately, no information is currently available on tissue expression and transcriptional regulation of TMX5. TMX5 possesses four putative N-glycosylation sites and one type-a TRX-like domain within its N-terminal luminal portion. The core of its TRX-like domain is represented by a non-canonical CRFS active site. The absence of the C-terminal cysteine residue within its active site defines TMX5 as a natural trapping mutant protein [57]. Indeed, the N-terminal cysteine of the TMX5 active site can nucleophilically attack free thiol group in protein substrates, but the interaction can be resolved only by the intervention of an external cysteine provided by another PDI or by the protein substrate itself. As such, the mixed disulfide between TMX5 and client proteins results stabilized and it could be long-living.

To date, no evidence has been reported on the physiologic roles of TMX5. Mutations in *TMX5* gene have recently been associated with the development of the Meckel-Gruber syndrome (MKS), a rare perinatally lethal autosomal recessive disease caused by defective ciliogenesis [58]. Deletions and missense mutations result in the generation of truncated forms of TMX5 that do not localize within primary cilium or periciliary regions as the wild type [59,60,61]. Thus, the mis-localization or the premature degradation of TMX5 might correlate with the onset of such ciliopathies. Indeed, it has been found that patients’ derived mutated fibroblasts as well as cells subjected to siRNA knockdown have a reduced number of ciliated cells, abnormal ciliary morphology and an aberrant localization to the transition zone of TMEM67 [59,60], a crucial regulator of cilia function [62].

## 7. Concluding Remarks

Among the more than 20 members of the PDI family, TMX1 represents the first example of topology-specific redox catalyst involved in both protein folding and ERAD pathways. Even though many details about its function have been established, we are still far from the complete knowledge of TMX1 dynamics and regulation. If the topology of client proteins represents a clear discriminant for the intervention of TMX1 in both folding and degradation and its participation in functional complexes with CNX explains its activity on N-glycosylated clients, no data are yet available about a possible participation of TMX1 in folding and/or degradation of non-glycosylated protein substrates. This information would give new insights on cargo selection and raise new questions about alternative binding partners that could drive and facilitate the recruitment of TMX1 clients. Most likely, the association with an alternative ER chaperone would determine a different sub-ER localization for TMX1, e.g., adjacent to the retro-translocation machinery to support the role of TMX1 in ERAD. In this scenario, TMX1 activity would be determined by the cooperative association with different ER-resident partners that would regulate the dual role of TMX1 in protein biogenesis or in protein clearance from the ER. Additional work is also needed for the dissection of the molecular dynamics behind TMX1 modulation of SERCA2b function: these data will be also instrumental to determine if TMX1 could be exploited as both diagnostic and therapeutic target for the treatment of select cancers.

For the other members of the TMX family, the scarce literature limits the available information on their function and regulation. Based on the current knowledge, TMX2 is the only member of the TMX family displaying its peculiar type-a TRX-like domain within the cytosol. This structural feature together with its SNDC active site could suggest that TMX2 is not directly involved in folding and degradation of secretory client proteins. However, it has recently been shown that cytosolic TRX-containing proteins are required for the formation of the correct disulfide bonds within the ER lumen [63]. For its function as calcium flux regulator, it is still not known if TMX2 targeting to MAM occurs via palmitoylation, as in the case of TMX1 [22]. Additionally, considering the structural differences between TMX1 and TMX2, it is likely that the two proteins would regulate calcium flux at the MAM via different mechanisms. As such, the dissection of the mechanistic behind these processes will help to establish if there is any crosstalk between TMX1 and TMX2, or if they rather act independently in different conditions or with different tissue specificity.

Among the five members of the TMX family, TMX3 is the only protein displaying an oxidase activity, as well as two b-type TRX-like domains. Interestingly, the type-a TRX-like domain of TMX3 displays the same active sequence of PDI, which is the master ER oxidoreductase acting during both protein folding and ERAD [64,65]. Thus, considering this property, TMX3 could be involved in the same processes. Since TMX3 and PDI have a different topology, being the latter soluble, it could be envisioned that TMX3 acts during both folding and ERAD of a select sub-group of client proteins. Moreover, the presence of two b-type TRX-like domains could suggest the engagement of different co-factors. Future studies exploiting a *trapping mutant* version of TMX3 would be important to characterize its functions in vivo and establish its potential partners and substrates specificity.

The *trapping mutant* approach would be instrumental also for the characterization of TMX4. Since it represents the N-glycosylated paralogue of TMX1, it will be interesting to compare these two members of the TMX family to dissect the reasons for their duplication, and in turn, to assess if there is a certain degree of functional redundancy. Considering their differences in tissue expression, it is possible that TMX1 and TMX4 exert the same functions within different tissues.

Another peculiar member of the TMX family is TMX5. The only information reported in the literature links TMX5 mutations to the development of a family of rare ciliopathies; however, the mechanisms behind this phenotype are still unknown. Starting from its peculiar active site, hypotheses can be formulated about its possible role in vivo. Indeed, the non-canonical *CRFS* sequence is shared by another member of the PDI family, ERp44 [66]. This well-known soluble protein is responsible for thiol-mediated protein quality control within the ER: the unique cysteine of its natural trapping mutant active site stably engages client protein substrates through their mispaired cysteine residues, mediating their efficient retention within the ER lumen and thus impeding the secretion of unfolded proteins intermediates [67]. As such, a parallel between ERp44 and TMX5 would allow the dissection of their possible functional similarities and substrates preferences.

In this review, we recapitulate the current knowledge about the features and roles of the members of the TMX family. These are five membrane-tethered PDIs, which are characterized by an ER signal sequence and a type-a TRX-like domain. Despite their similarities, the TMXs also show some structural differences, which could hint at a certain degree of functional diversification and specialization among the different members of the TMX family. Future dissection of their individual roles are needed to enlarge our knowledge about PDIs functions, and to allow the comparison between the members of the same TMX family and between membrane-tethered and soluble PDIs.

## Figures and Tables

**Table 1:** List of the TMX family members. The table displays the main features of the five TMXs including their active site sequences and biological functions. a, active type-a TRX-like domain; b, inactive type-b TRX-like domain; R, reductase activity; O, oxidase activity.

Protein	TRX-Like Domains	Active Site	Activities	Biological Functions
TMX1	a	CPAC	R	Protein folding and ERAD Ca^2+^ flux regulation
TMX2	a	SNDC	?	Nuclear protein import Ca^2+^ flux regulation
TMX3	abb’	CGHC	O	?
TMX4	a	CPSC	R	Protein folding
TMX5	a	CRFS	?	?
